# Near-source wastewater surveillance as a non-invasive tool for disease detection in prisons

**DOI:** 10.1038/s41598-026-35801-1

**Published:** 2026-01-31

**Authors:** O. O’Mara, F. Hassard, K. Jobling, M. Quintela-Baluja, S. McIntyre-Nolan, L. Lundy, A. C. Singer, S. Rahimzadeh, I. Stanton, V. Castro-Gutierrez, H. Charlotte-Smith, M. Vu, R. Pedley, P. Adamou, D. W. Graham, M. Di Cesare

**Affiliations:** 1 University of Essex, Colchester, UK; 2 Cranfield University, Cranfield, UK; 3 Newcastle University, Newcastle upon Tyne, UK; 4 Durham University, Durham, UK; 5 Middlesex University, London, UK; 6 UK Centre for Ecology and Hydrology, Wallingford, UK; 7 Environmental Pollution Research Center (CICA), University of Costa Rica, San Pedro, Costa Rica; 8 Cranfield Water Science Institute, Cranfield University, Cranfield, UK

**Keywords:** Diseases, Environmental sciences, Microbiology

## Abstract

Near-source wastewater-based epidemiology (WBE) offers a non-intrusive alternative to clinical testing of whole prison populations. Prisons sit at the centre of high transmission risk but experience limited health-care access and barriers to testing individual prisoners. However, the use of WBE for health protection in prison settings has been limited. To assess its merit during the COVID-19 pandemic, SARS-CoV-2 RNA concentrations were quantified in 680 composite wastewater samples collected from 14 prisons across England and Wales between January and June 2021. Viral RNA was detected in 48% of samples, and wastewater viral loads were found to closely mirror clinical case numbers Lead–lag analysis with adjacent municipal wastewater samples indicated a bidirectional flow between the prisons and their local community: seven prisons exhibited wastewater peaks ahead of their communities, while six lagged, highlighting heterogeneous epidemiological coupling. Marked differences between prisons were apparent in both physicochemical wastewater traits and clinical testing uptake, indicating each institution constitutes a distinct surveillance unit. Collectively, findings here indicate near-source WBE as a rapid, unbiased and scalable tool for disease outbreak detection and for mapping disease flow between prisons and their surrounding communities, advocating its integration into routine health-security frameworks for custodial and other high-density settings.

**Supplementary Information:**

The online version contains supplementary material available at 10.1038/s41598-026-35801-1.

## Introduction

Individuals incarcerated in prisons are among the most marginalised and excluded groups of society^1^, facing significant public health risks^2,3^. In prisons, often marked by overcrowding, poor hygiene, and unsanitary conditions^4^, prisoners reside in almost “perfect habitats” for the spread of airborne and other diseases^5^. Combined with generally poor health profiles, prisons tend to have much higher prevalence of multimorbidity, chronic illnesses, and infectious disease outbreaks compared with wider communities^6,7^.

During the COVID-19 pandemic, prison residents (i.e., prisoners) experienced higher rates of infection, hospitalisation, and death^8,9^ than national averages. For example, during the first COVID-19 wave in England and Wales in 2020, prisons recorded 7.6 confirmed COVID-19 cases per 1000 people compared with only 4.9 cases per 1000 people in the general population^10^. In January 2021, COVID-19 case rates in prisons were approximately three times greater than in the wider community, with over 1000 daily cases reported at that time in the UK^9^.

Such adverse health outcomes reflect broader health inequalities experienced by prisoners^11^. Prisons have a long history of infectious disease outbreaks, including tuberculosis, influenza (types A and B), varicella, measles, mumps, viral hepatitis and adenovirus^12,13^. Additionally, prisoners are more likely to have experienced homelessness^14^, substance misuse^15,16^, and mental health issues^17^. Various studies have highlighted the relationship between social factors and infectious disease^18–20^, emphasising the overlap between the social determinants of health and crime. However, the burden of disease in prisons is not solely due to their closed nature, as some infectious diseases easily transmit “through the bars” in a prison^21^, with prisoners and staff regularly moving between custody and the wider community. Prisons are rarely closed systems^22^, but the extent to which they ecologically interact with wider society remains unclear.

Wastewater-based epidemiology (WBE) is an increasingly utilised approach for public health surveillance, providing a less biased estimate of disease prevalence at community scales than clinical testing^23^. WBE involves the quantification of biochemical signatures detectable in wastewater (including human urine and faeces) to offer insights into population health, behaviours, exposures, and interventions^24^. Previous studies have successfully applied WBE approaches to detect COVID-19^25^, antimicrobial resistance (AMR)^26^, influenza^27^ and other viral pathogens^28^ to estimate community-level trends in disease. Such studies include monitoring at near-source settings, such as schools^29^, hospitals^30^, aircraft^31^, airports^32^, and cruise ships^33^. While WBE is now an established public health surveillance tool, its regular use in prisons remains under-explored, despite unique health risks among their residents.

Existing prison research on WBE has predominantly focused on monitoring illicit drug consumption and pharmaceutical misuse^34^ and demonstrated that wastewater analyses provide more comprehensive data on drug use frequency compared to random urinalyses alone^35^, offering prison administrators valuable insights into substance abuse patterns and intervention effectiveness. However, a significant research gap exists regarding the utility of WBE for infectious disease surveillance in prison populations. While there are some qualitative studies^36^ and case studies^37,38^, few have examined the temporal relationship between clinical cases and wastewater monitoring between prison and community data in England, representing an important opportunity for public health advancement.

Here, we examine the epidemiological relationship between SARS-CoV-2 in prisons and their surrounding communities, using WBE data as a guide. Epidemiologists seek to identify patterns and trends in disease occurrence by analysing environmental, behavioural, and genetic factors^39^. However, traditional methods are limited to known exposures. Monitoring methods are inhibited further in prisons because of a lack of resources for testing and general prisoner distrust towards state interventions and isolation^9^. This study aims to (1) assess the effectiveness of near-source WBE in detecting SARS-CoV-2 in prison settings, (2) explore possible epidemiological connections between prisons and their surrounding communities, and (3) evaluate the potential of WBE to reduce health inequalities and monitor disease in vulnerable populations. Using SARS-CoV-2 wastewater monitoring and COVID-19 case data from 14 prisons in England over six months in 2021, we indicate how WBE-based monitoring, combined with other interventions, can enhance health protection in prisons, particularly in enabling the control of infectious disease transmission in these high-risk settings.

## Results

From January to June 2021, 680 wastewater samples were collected from 14 prisons (Table 1) to quantify SARS-CoV-2 RNA concentrations across the prisons. Among these prisons, three were category A (the highest security level in the UK), two were category B (local prisons receiving directly from the court), seven were category C (training facilities for lower escape risk prisoners), and two were female-only prisons. All of our sample sites were ‘closed’ prisons, meaning that prisoners spent all their time inside the prison.

**Table 1:** Sample sites and prison demographics.

Prison	Function	Security category	Prisoner population (as of June, 2021)^[1]^	Median age of prisoner pop. (June, 2021)	Workforce population (Full-time equivalent as of June, 2021)^[2]^	Number of Operational (prisoner-facing)	Number of Non-operational (non-prisoner facing)
PBX	Resettlement	C	650	35.21	535	436	99
PNT	Trainer & Resettlement	C	1300	37.02	600	n/a	n/a
PLN	Local & Resettlement	Female	250	37.02	250	200	50
PHH	Trainer & Resettlement	C	1000	39.13	500	400	100
PFK	Trainer	A	800	42.15	800	700	100
PDHA&B	Reception/ Local	B	900	33.7	400	350	50
PDB	Trainer	C	250	18–20	250	200	50
PWF	Trainer	A	700	56.22	550	450	100
PNH	Local & Resettlement	Female	350	35.14	300	200	100
PML	Trainer & Resettlement	C	900	36.48	400	287	100
PLH1& 2	Trainer	C	900	35.35	400	300	100
PHB	Trainer & Resettlement	C	900	35.46	431	325	106
PHO&N	Reception/ Local	B	1000	35.11	455	356	99
PFS	Trainer	A	550	40.79	592	506	86

[1]Figures rounded to nearest 50 to protect anonymity. Available via: HMPPS (2021, October) Offender Management Statistics quarterly: April to June 2021.

[2] Figures rounded to nearest 50 to protect anonymity. Available via: HMPPS (2021, August) Her Majesty’s Prison and Probation Service workforce quarterly: June 2021.

Security category: A - High-security prisons. They house male prisoners who, if they were to escape, pose the most threat to the public, the police or national security; B - Local or training prisons. Local prisons house prisoners that are taken directly from court in the local area (sentenced or on remand), and training prisons hold long-term and high-security prisoners; C - Training and resettlement prisons; most prisoners are located in a category C. They provide prisoners with the opportunity to develop their own skills so they can find work and resettle back into the community on release; Female - closed women-only prison. Women are held in either open or closed conditions.

The prison populations ranged from 250 to 1300 prisoners, with an average of 746.4 prisoners per prison. The median age of the prisoners varied between 33.7 and 56.2 years (with one prison (PDB), reporting age as categorical). The full-time equivalent workforce in each prison ranged from 250 to 800 employees, averaging 461.6 employees per prison. Of these, the operational workforce (prisoner-facing) ranged from 200 to 700 employees, with an average of 362.2 employees per prison. Each prison had distinct daily operational procedures, cleaning schedules, movements, prisoner and staff profiles, and physical structures.

Lastly, all participating prisons remained ‘open’ throughout the pandemic ‘to serve the courts’^40^, and in most prisons, prisoners and staff interacted with the wider community through day releases, hospital visits, social visits, court attendance, and via staff turnover. None of the prisons were wholly isolated from their host community.

### The relationship between prison wastewater and prison clinical testing

Wastewater samples were collected up to four times per week from each prison for 12 weeks. Except for PBX, viral RNA fragments were detected in all prison sewers during the monitoring programme (Appendix Fig. 1, Appendix Table 1). SARS-CoV-2 amplicons (N1 and/or E genes) were detected in 48% (*n* = 329) of samples, of which 68% (*n* = 225) tested positive for both N1 and E genes, 20% (*n* = 65) for only N1 and 12% (*n* = 38) for only E gene. Across all prisons, the average concentration detected by RT-qPCR was 1.07 × 10^6^ GC/L for N1 and 9.42 × 10^9^ GC/L for E. The lowest detected concentrations for N1 and E were 1.11 × 10^2^ GC/L and 2.67 × 10^2^ GC/L, respectively; whereas the highest levels were 2.11 × 10^8^ GC/L for N1 and 2.54 × 10^11^ GC/L for E.

**Fig. 1:**
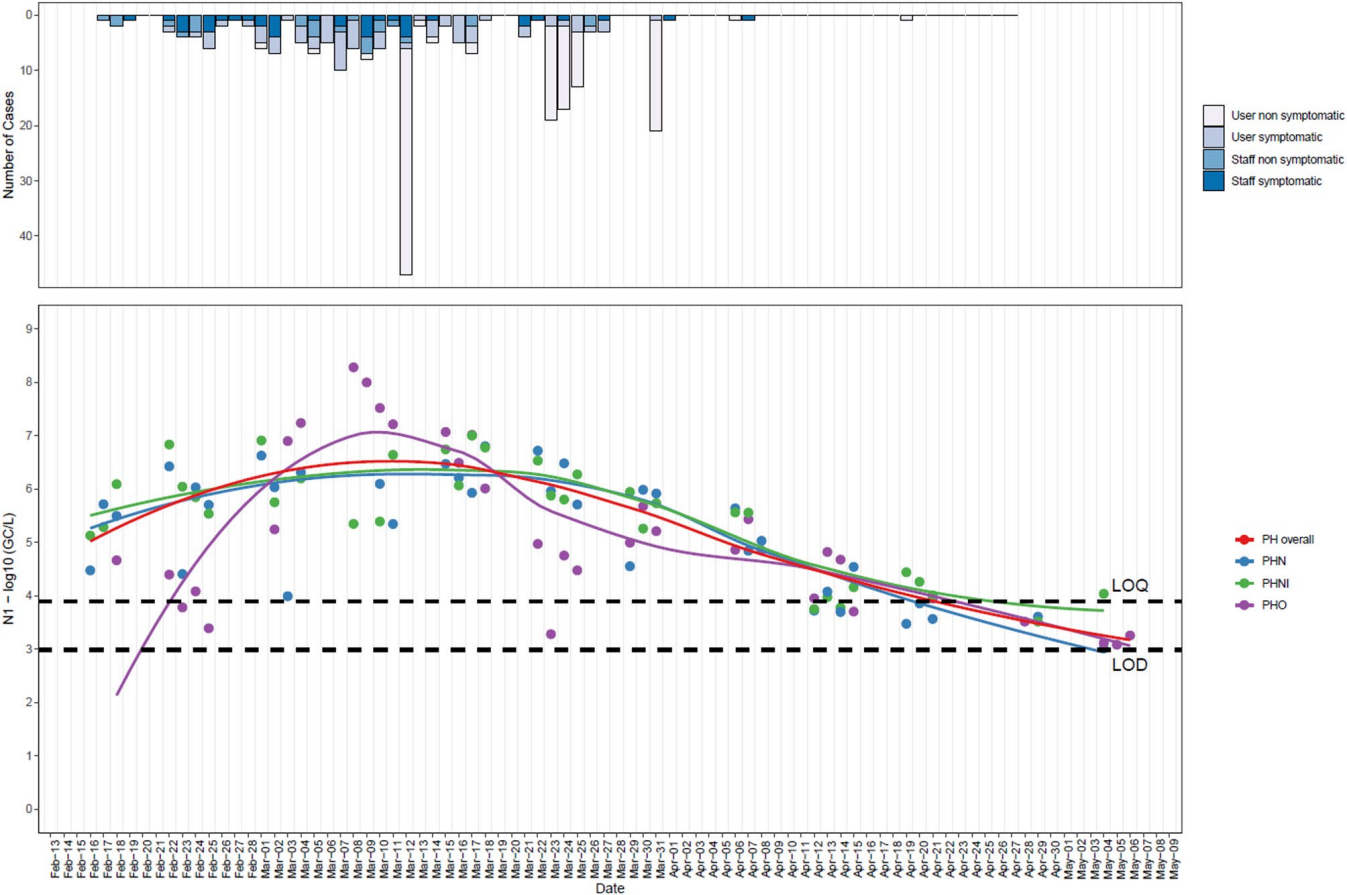
Relationship between COVID-19 clinical cases and wastewater viral loads. Top Panel: Daily number of new COVID-19 cases. Targeted mass testing on March 12 and comprehensive mass testing across the entire prison on March 23, 24, 25, and 31. Bottom Panel: SARS-CoV-2 N1 gene segment concentrations (log10 genomic copies per litre, GC/L) in wastewater. Each point represents the log-transformed concentration from two sampling sites (PHN and PHO) collected overnight and a high-frequency sampling site (PHNI) with samples collected every 5 min from 07:00–09:00. The LOESS (Locally Estimated Scatterplot Smoothing) regression lines depict trends for each site: blue for PHN, purple for PHO, and green for PHNI, with the overall trend shown in red. LOQ: limit of quantification; LOD: limit of detection.

In response to increasing SARS-CoV-2 levels detected in prison wastewater, two prisons (PHO&N and PML) launched targeted and mass testing among their prisoners. On 12 March 2021, PHO&N commenced clinical mass testing (PCR nasal and throat swabs on 83% of the prison population) followed by comprehensive mass testing across the entire prison on 23, 24, 25, and 31 March 2021. Conversely, PML organised two mass testing events on 1 April and 8 April 2021.

**Fig. 2:**
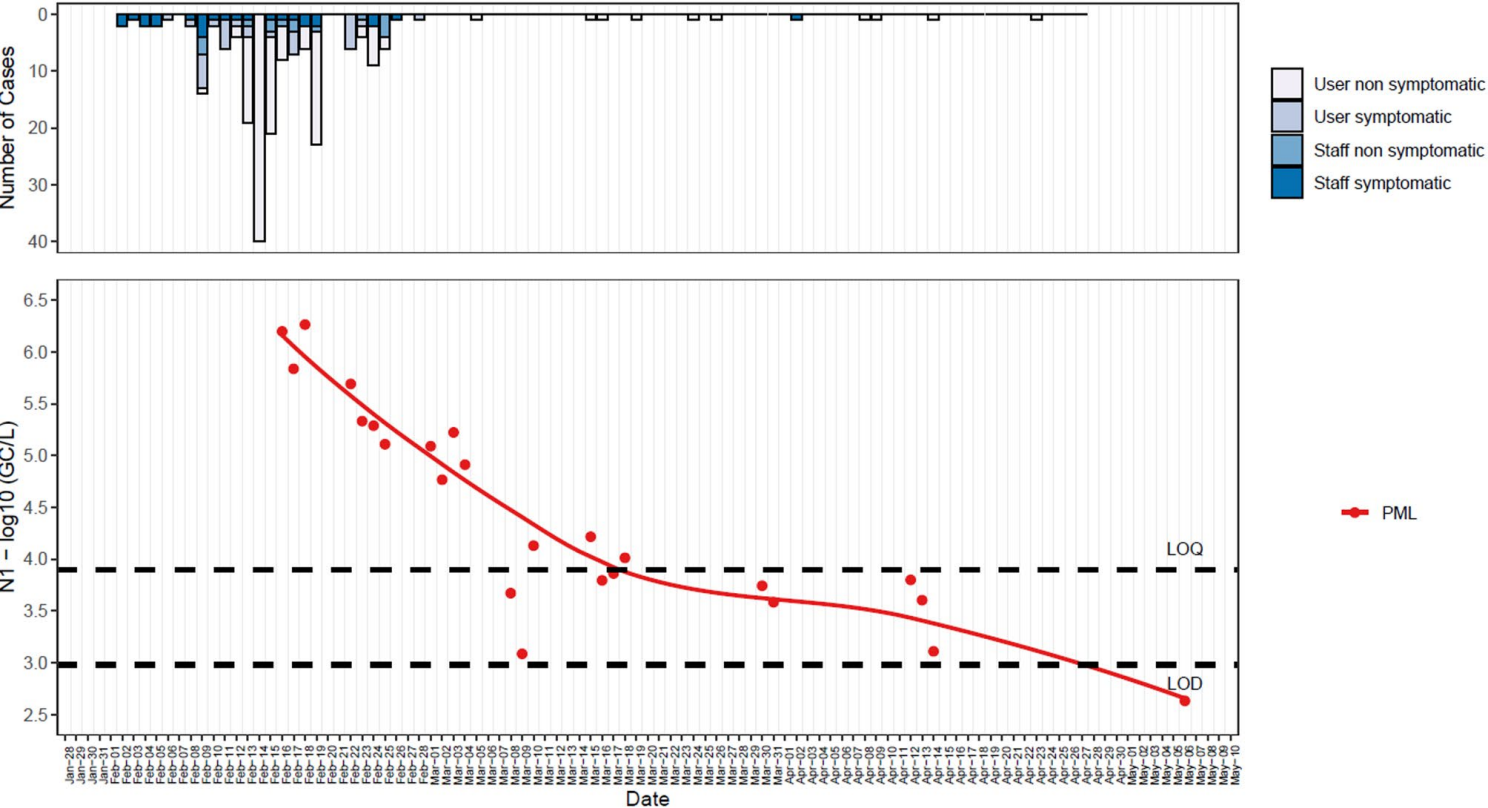
Association between COVID-19 case counts and wastewater viral loads during the declining phase of the outbreak at PML. Top Panel: Daily number of new COVID-19 cases. Mass testing events on April 1 and April 8 2021. Bottom Panel: SARS-CoV-2 N1 gene segment concentrations (log10 genomic copies per litre, GC/L) in wastewater. Each point represents the log-transformed concentration from PML. The LOESS (Locally Estimated Scatterplot Smoothing) regression lines depict trends. LOQ: limit of quantification; LOD: limit of detection.

SARS-CoV-2 detection in wastewater confirmed a strong association between clinical cases and wastewater concentration levels during the peak of the outbreak at PHO&N (ρ = 0.728, *p* < 0.001) (Fig. 1) and at the end of the outbreak at PML (ρ = 0.409, *p* < 0.001) (Fig. 2).

**Fig. 3:**
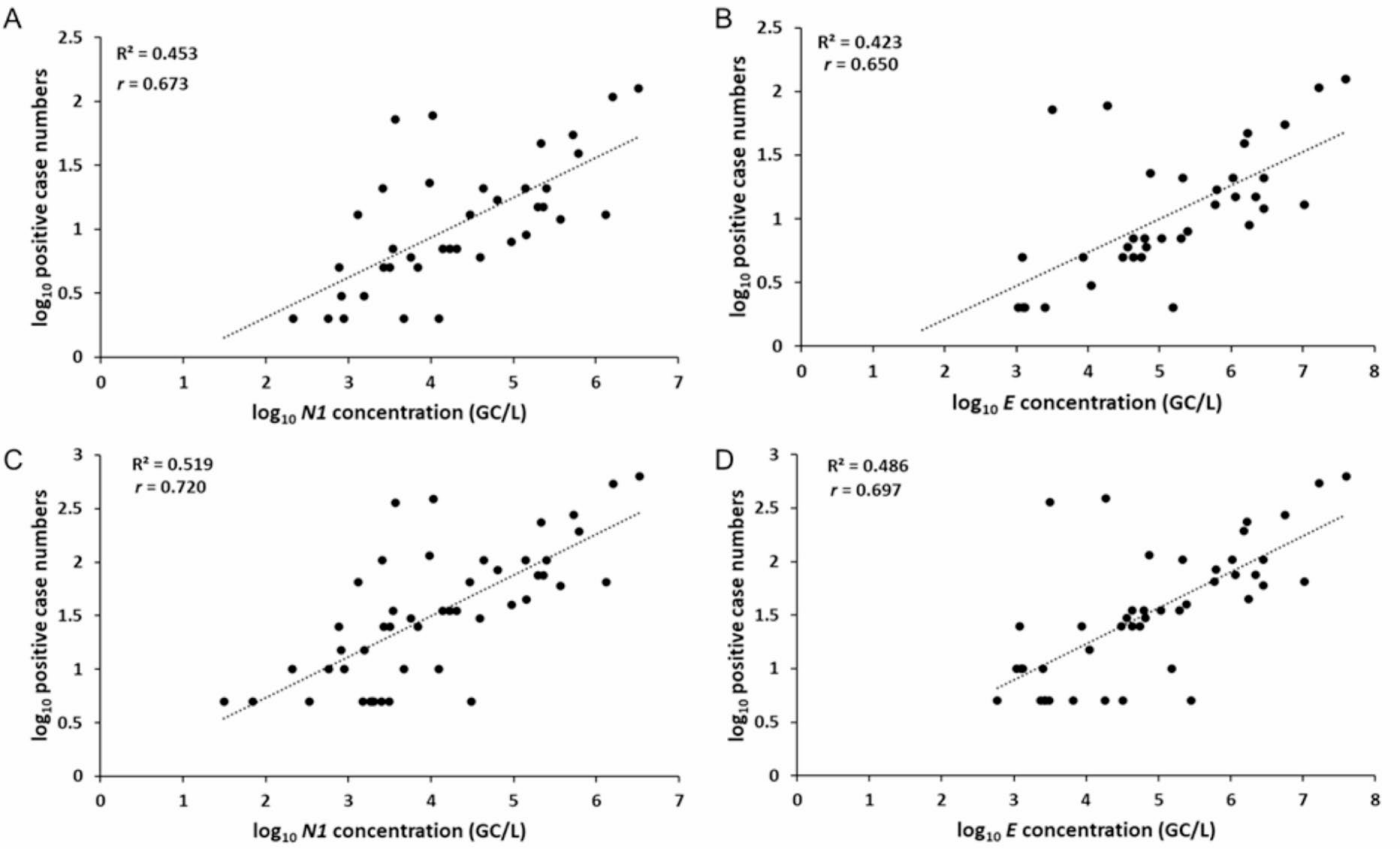
Correlation between weekly clinical cases and SARS-CoV-2 wastewater concentrations. Relationship between positive COVID-19 case numbers (excluding mass testing data) and wastewater concentrations of SARS-CoV-2 *N1* and *E* genes (GC/L). Panels A and B consider log transformed weekly case data and average weekly wastewater concentrations. Panels C and D consider corrected case numbers considering the 5-fold under-reporting ratio calculated through mass testing data. Correlation measure with Spearman’s rank correlation coefficient (r).

Although mass testing data were unavailable for all monitored prisons, testing did occur on symptomatic users, their close contacts and via voluntary staff testing throughout our sampling period. Testing uptake among staff and prisoners was highly variable across the prisons^9^, but it was still possible to make comparisons between available weekly COVID-19 case data and average weekly SARS-CoV-2 levels detected in wastewater at the 12 prisons that did not have mass testing (a “week” is defined as the first sampling day, Monday, and the subsequent 6 days). A positive association between case numbers and SARS-CoV-2 concentrations in wastewater for both N1 (ρ = 0.673, *p* < 0.001) and E (ρ = 0.650, *p* < 0.001) was detected (Fig. 3 panels A and B, and Appendix Table 1 for prison-specific results). Specifically, during the targeted clinical mass testing in response to wastewater signals at PHO&N on 12 March 2021, 45 COVID-19 positive cases were confirmed. This was 5-fold and 6.1-fold higher than the pre- and post-7-day average case numbers based on the ad-hoc clinical testing strategy. When case numbers at prisons which did not undergo mass testing were corrected using the 5-fold under-reporting ratio, these correlations increased to ρ = 0.720 (*p* < 0.001) and ρ = 0.697 (*p* < 0.001) for N1 and E, respectively (Fig. 3 panels C and D).

Overall, COVID-19 outbreaks and infection patterns were accurately detected using virus data from wastewater analysis. WBE data accurately tracked SARS-CoV-2 trends, and correlations between clinical cases and wastewater data were established to guide local health protection responses. This confirms the value of wastewater monitoring in outbreak management within prisons. Wastewater monitoring was effective in identifying outbreaks early and informing the impact of public health interventions, as evidenced by fewer cases in PHN&O following the introduction of improved public health measures.

### Prisons as potential indicators of community disease outbreaks

To understand the degree of epidemiological connection between the prisons and their local communities, a lead/lag analysis was performed comparing concentrations of SARS-CoV-2 found in prison wastewater to those in the surrounding community in which the prison was located. Two prisons, PNT and PDB, were not in proximity to suitable community wastewater sampling locations and due to the late inclusion of PBX in the study, these prisons were omitted from the analysis.

**Fig. 4:**
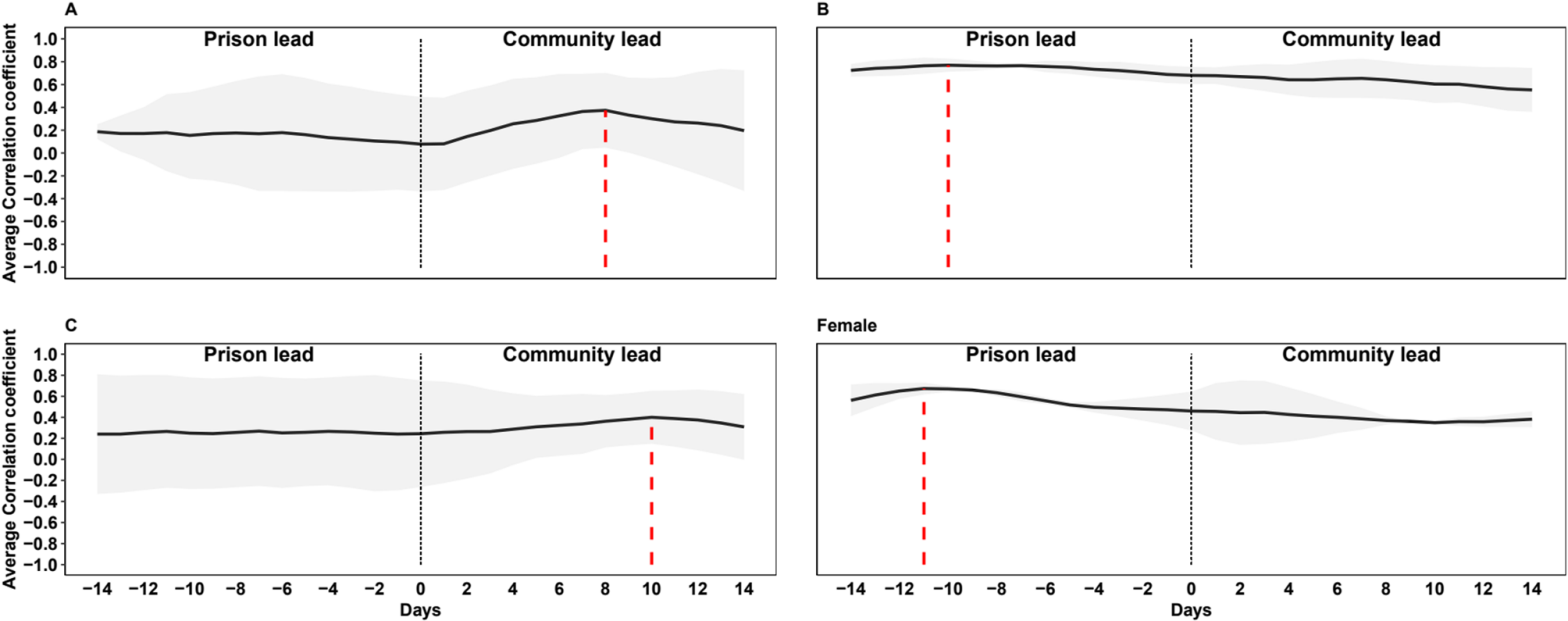
Lead/lag analysis of SARS-CoV-2 concentration levels in prison and community wastewater by type of prison. (**A**) High-security prisons; (**B**) Local or training prisons; (**C**) Training and resettlement prisons; Female - closed women-only prison. The grey shadow represents the standard deviation. The dashed red line indicates the time when the correlation coefficient is highest.

Based on all 13 sites, concentrations of SARS-CoV-2 in community samples preceded prison samples by about 8 days (ρ = 0.43), although the relationship was not statistically significant (*p* = 0.43) (Appendix Fig. 2). However, different patterns were identified at the prison level, with the SARS-CoV-2 viral load leading to detection in community wastewater in some sites and lagging in others (Appendix Fig. 3). The analysis by security categories (Fig. 4) highlights how for prisons category A and C, community concentrations tended to lead prison concentrations respectively by about 8 and 10 days. On the contrary, for category B and only-women prisons, concentrations led community concentrations respectively by approximately 10 and 11 days.

### Prisons physicochemical profile

Physicochemical analysis of prison wastewater also revealed how different prisons tended to be distinct from each other, specifically conductivity (EC), pH, ammonia (NH₄-N), and total suspended solids (TSS) levels (Appendix Table 2). Data showed high variability both between prisons and within samples from the same facility. The mean values (ranges in brackets) for EC, pH, NH₄-N and TSS across the prisons were 1624 µS/cm (8–8,795 µS/cm), 7.72 (5.44–10.25), 43.4 mg/L (8–118 mg/L) and 232 mg/L (8–14,180 mg/L).

**Table 2:** Mean, minimum and maximum concentrations for each sample site (N1 and E gene targets).

Prison	Mean N1 (GC/L)	Min N1 (GC/L)	Max N1 (GC/L)	Mean E (GC/L)	Min E (GC/L)	Max E (GC/L)
PBX	ND	ND	ND	ND	ND	ND
PNT	1.64E + 05	2.64E + 02	1.79E + 06	6.60E + 05	7.28E + 02	3.67E + 06
PLN	8.97E + 03	3.11E + 02	4.69E + 04	1.18E + 05	3.01E + 02	5.49E + 05
PHH	1.44E + 05	1.29E + 02	6.84E + 05	1.00E + 06	3.84E + 02	5.68E + 06
PFK	ND	ND	ND	ND	ND	ND
PDHA	8.96E + 05	1.51E + 02	9.07E + 06	9.42E + 09	7.72E + 03	2.54E + 11
PDHB	8.81E + 05	1.45E + 03	1.17E + 07	6.16E + 06	3.90E + 03	1.12E + 08
PDB	ND	ND	ND	2.58E + 03	1.47E + 02	5.02E + 03
PWF	6.86E + 03	1.11E + 02	4.03E + 04	4.59E + 04	5.56E + 02	4.36E + 05
PNH	8.58E + 03	5.36E + 02	6.90E + 04	1.35E + 05	4.20E + 02	5.96E + 05
PML	2.85E + 05	4.29E + 02	1.84E + 06	3.08E + 05	8.30E + 02	1.98E + 06
PLH1	1.44E + 03	7.93E + 02	2.27E + 03	2.93E + 03	2.93E + 03	2.93E + 03
PLH2	2.80E + 03	1.11E + 02	1.13E + 04	ND	ND	ND
PHB	8.46E + 03	2.12E + 02	3.94E + 04	1.16E + 04	4.38E + 02	3.61E + 04
PHN	1.12E + 06	1.02E + 03	7.77E + 06	1.55E + 06	2.67E + 02	8.58E + 06
PHO	1.15E + 07	1.20E + 03	2.11E + 08	8.63E + 06	1.32E + 03	1.18E + 08
PFS	1.69E + 03	1.22E + 02	3.04E + 03	3.79E + 03	3.79E + 03	3.79E + 03

In PLH (PLH1 and PLH2), PDH (PDHA and PDHB) and PH (PHO and PHN) samples were collected from two different collection points.

When data from all prisons were combined, significant positive correlations were observed between N1 gene concentrations and conductivity (*r* = 0.2874, *p* < 0.0001) and NH₄-N (*r* = 0.2468, *p* = 0.0047), respectively. Similarly, E gene concentrations also correlated significantly with conductivity (*r* = 0.1247, *p* = 0.0429) and NH₄-N (*r* = 0.2040, *p* = 0.0126) as well as pH (*r* = 0.1701, *p* = 0.0056). However, at the individual prison level, correlations varied widely; some prisons showed significant relationships between certain parameters and viral gene concentrations, while others did not.

This inconsistency underscores the uniqueness of each prison, influenced by factors such as management practices, size, and population dynamics. These findings highlight that wastewater characteristics are strongly influenced by the specific conditions of each facility, emphasising the need for tailored surveillance strategies for each prison or security category of prison.

Wastewater analysis further identified variants prevalent at different phases of the pandemic. Analysis during February and March 2021 revealed that the dominant variant circulating was Alpha. This is evidenced by the high (> 75% in all cases) allele frequency of key mutations associated with the Alpha variant. Notably, our genomic analyses also detected the emergence of Delta AY.1 (B.1.617.2_K417N) in a subset of prison wastewater samples. Genomic surveillance data from the UK Health Security Agency indicates that the Delta variant (B.1.617.2) overtook the Alpha variant and became the dominant circulating strain around mid-June 2021—specifically, epidemiological evidence points to approximately 12 June 2021 as the point when Delta accounted for over 50% of sequenced cases. This marked a significant epidemiological shift in the UK’s SARS‑CoV‑2 landscape. The concurrent detection of Delta AY.1 in prisons occurred as it was emerging in the community, suggesting that prison environments are not epidemiologically isolated, although lead-lag data suggest the local flow of such disease varies from place to place. Broadly speaking, findings here imply that prisons mirror broader community trends in variant emergence, reinforcing the public health value of wastewater monitoring in these settings to detect and track emerging Variants of Concern or under Investigation.

## Discussion

Here, we explore the epidemiological relationship between prisons and wider communities within the context of infectious disease. Existing social and cultural interpretations of imprisonment describe prisons as a ‘total institution’^41^, self-sustaining and all-encompassing environments with minimal interaction beyond their walls. Alternatively, prisons have been characterised as ‘permeable’ and ‘porous’^5^, highly interconnected with wider society. Our findings contribute to the ongoing debate on the role of prisons in public health, highlighting the types of connections to the outside world and heightened vulnerability of prison residents to infectious disease outbreaks.

Disease patterns within and between prisons may differ from those in the wider community because of local procedural differences, yet prisons can act as both the source and recipient of outbreaks^42^ due to their endemicity (persistence of disease presence)^43^, design (amplification and reservoir/ founder effect of disease)^44^, and demographics (susceptibility, hard to reach populations, deprivation)^45^. Therefore, precise, consistent and robust surveillance systems are essential for effective disease preparedness, prevention, detection and response in these settings.

By *precise*,* consistent*,* and robust surveillance systems*, we refer to approaches that can generate accurate and reliable health signals from prison populations in a way that is both actionable and sustainable. *Precision* denotes the analytical accuracy required to detect disease presence at low prevalence and to distinguish real changes in infection patterns from background variability. *Consistency* refers to the ability to deliver data with sufficient reliability and timeliness to give operational partners confidence to act quickly, whether in initiating outbreak control, targeting testing, or adjusting prevention measures. *Robustness* reflects the capacity of the system to withstand both scientific and institutional challenges: it must be resilient to analytical scrutiny, as well as operational barriers such as funding constraints, sewer blockages, prisoner behaviours (e.g. ragging), or damage to infrastructure. A robust system should also be able to track population health trends over time, capturing both acute outbreaks and underlying endemicity, without being dependent on high levels of human resources to maintain its functioning. In this sense, wastewater-based surveillance offers a scalable and sustainable platform for health protection in prisons, complementing and enhancing existing clinical surveillance systems.

Our analysis provides various insights for health protection in prisons. Foremost, wastewater surveillance conducted near the source at penal institutions is a reliable and accurate method for ambient disease detection. This approach has shown its effectiveness in prisons^46^, where traditional clinical testing is often hindered by resource limitations, symptom concealment, and uncontrolled transmission^47^. Wastewater provides a ‘measure once – test (for) many’^29^ strategy, enhancing the efficiency of data gathering, increasing public health insights per sample, and tracking intervention effectiveness over time.

Guided by One Health principles, 14 UK prisons were monitored for SARS-CoV-2 using wastewater analysis, offering a less disruptive yet highly effective tool for public health intervention. This near-source approach overcomes institutional barriers to clinical testing and enables a precise response to the dynamic trends of pathogen prevalence, which, during the COVID-19 pandemic, precipitated enhanced testing and isolation protocols in both custody and the wider public. Beyond infectious disease containment, WBE extends its utility to the surveillance of other faecally excreted pathogens and various chemicals and pharmaceuticals^34^, thereby potentially informing tailored response measures.

Compared to mass clinical testing, WBE is a cost-efficient and fast population-level indicator that is less susceptible to healthcare-seeking and test-access biases than case-based surveillance. Sensitivity is constrained by assay detection limits, matrix-derived PCR inhibition, and sampling design.h. Even when prisoners consent to clinical testing, testing whole populations (‘mass testing’) was ‘extremely burdensome to healthcare teams and compromise(d) their ability to deliver business as usual and to recover from the pandemic healthcare backlog’^9^. Mass testing was generally considered complicated, time-consuming and labour-intensive. Near-source WBE offers a practical alternative for continuous population surveillance in carceral settings^48^. As Yoo et al. (2023) identified in their cost-benefit evaluation in Japan, wastewater surveillance is more economical than clinical screening options at low incidence^49^. This wastewater-based surveillance strategy alleviates pressure on healthcare resources while improving the health and safety of incarcerated individuals and, by extension, local communities.

Wastewater monitoring is considered more socially acceptable for the target population. Sociological research indicates that incarcerated individuals largely prefer wastewater surveillance over clinical testing due to reduced personal invasion, indicating greater potential for acceptance within prison communities^50^. Such observations were seen during the pandemic, where some prisoners refused clinical testing to avoid isolation, but were more accepting of anonymised passive surveillance, such as WBE^9^. One USA-based study highlighted that prisoners generally preferred WBE monitoring as a less intrusive and more reliable surveillance strategy^51^. Our qualitative experience of explaining the research to prisoners, whilst unquantified, reinforces these findings. Near-source WBE overcomes many procedural and personal barriers to monitoring prisoner health.

WBE offers a valuable alternative to inconsistent clinical testing practices. Mass testing was often limited in its epidemiological insights as it was a snapshot of the consenting population at one time. As a surveillance method, clinical testing is unable to capture variable shedding among the population, is reliant on test sensitivity, and cannot be monitored for changes over time. It is valuable for identifying specific infected individuals, but wastewater monitoring provides a more useful tool for measuring SARS-CoV-2 concentration levels (potentially including variants) and other pathogens over time. These findings support extant international research^37^, validating the accuracy of wastewater monitoring for identifying cases in prisons. It facilitates long-term, longitudinal monitoring that spans weeks to years, supplying fast, actionable and accurate insights into pathogen dynamics.

A key strength in WBE is its immunity to selection bias. Unlike classical approaches, which depend on individual participation and lack demographic representativeness, WBE serves as an unbiased and comprehensive measure of community health, enabling timely interventions that could reshape public health strategies, particularly in closed and controlled environments. Although this depends on practitioner knowledge and acceptability^36^, our findings confirm that near-source WBE is a reliable tool for early detection of infectious diseases and continuous monitoring in prison settings. Where public health practitioners are trained and understand the utility of wastewater monitoring, it is an accurate and reliable indicator of community health.

WBE highlights the importance of prisons in a public health emergency. This analysis indicates that prisons have an epidemiological relationship with their local community, but are epidemiologically distinct from each other. We identified variability between prisons, possibly driven by demographic differences, behaviour differences, such as when prisoners excrete, and cultural differences, such as how prisoners and staff interact. This could also be affected by the purpose of the prison. For example, a high-security prison with a more stable population may have less interaction with its local community than a local category B prison that directly takes prisoners from court and has a higher turnover of new prisoners. Relatedly, on average, male prisoners stay three times longer in a high-security prison than in a local prison. Imprisoned men also stay longer in a prison (87.25 days mean time) than imprisoned women (74.1 days)^52^. The data does not reveal a clear explanation for the relational differences between prisons and their localities, but indicates that infection can be seeded into prisons from the wider community, from which the prison design can have an amplification effect. This supports extant findings that the physical and social design of prisons^5,43,53^ can quickly multiply a single case into many.

Monitoring these amplified prison outbreaks in wastewater may provide an early warning signal of community cases. Prisons are deeply interconnected with society through staff, visitors, and prisoners^54^, and near-source wastewater surveillance can help monitor these interactions. Wastewater surveillance of SARS-CoV-2 identified large and rapid outbreaks within prison populations, including the presence of variants. This monitoring identified connections between prisons and surrounding communities, where prisons may have preceded or contributed to community outbreaks, while also experiencing outbreaks linked to external sources. In contrast to qualitative studies that rely on practitioners’ subjective interpretations of case data^36^, we indicate how wastewater data can inform public health interventions, such as identifying infection trends, viral mutations, and evaluating the effectiveness of control measures earlier than clinical methods.

During the study, concentration detected (presence and trend) in prisons was provided weekly to each prison’s management and local public health practitioners to enable appropriate monitoring, management, and response, such as targeted clinical testing or outbreak testing of prisoners. In addition, the results were included in the SAGE report to support the UK Government’s response to COVID-19 transmission in prison settings^9^. WBE is a critical population monitoring tool to identify and reduce outbreaks in custody in England and Wales^9^.

In conclusion, this work demonstrates that WBE is an accurate tool for early outbreak detection in prison settings, providing a reliable alternative to individual clinical testing for tracking disease presence and trends. When combined with policy measures, wastewater surveillance approach, in conjunction with policy measures, offers vital health protection and helps mitigate the impact of infectious diseases in vulnerable environments. Our findings support the integration of WBE into routine health surveillance in prisons, enabling earlier interventions and reducing transmission. However, it is critical to acknowledge that each prison is a distinct social space and there is unlikely to be a ‘one-size-fits-all’ public health approach to outbreak management across different institutions. These insights support extant literature for exploring the application of WBE in other ‘total institutions’, including military barracks^55^, University halls^56^, hotels and immigration facilities^57^. A similar approach could also be beneficial in other high-density environments with restricted mobility, such as residential care homes^58^. Wastewater-based epidemiology is a solution to problems of population monitoring.

## Materials and methods

### Ethical approvals selected and prison selection

Ethical approval was granted by Newcastle University, Cranfield University and approved by HM Prison & Probation Service’s National Research Committee. Thirteen facilities in North East England (6) and Yorkshire & Humber (7) regions were selected. Each prison was monitored for up to 12 weeks between January 24, 2021, to June 23, 2021. One additional facility located in London was incorporated in April 2021 at the request of Public Health England (now the UK Health Security Agency) to identify a possible Variant of Concern. Prisons studied were selected to represent diversity in terms of function, size and security classifications (Table 2).

### Sample collection

​​Sample collection in each prison occurred daily from Monday to Thursday using an Aquacell P2-COMPACT (Aquamatic) autosampler installed in the main sewer line to represent samples from the whole prisoner population. In two facilities (PLH (1&2) & PHO&N) with distinct sections, dual sampling points were installed. Metadata were recorded for each sample collected, including location, date, time, and GPS coordinates. Following consultation with operational staff and prisoners, we undertook a detailed assessment of the prison regime to identify the periods when wastewater inputs would best reflect prisoner activity. This included understanding when potential contaminants, such as laundry or blockages (‘ragging’), were most likely to enter the system, and distinguishing the times of day when staff and prisoners typically used toilet facilities. From this operational mapping, a decision was made to reduce the composite sampling window to 14 h (19:00 to 09:00), targeting the period when the majority of prisoners used the facilities and staff use was minimal. This approach reduced extraneous contributions and maximised representativeness of the prison population, effectively minimising and in most cases eliminating the possibility of staff usage ‘contaminating’ the samples. A 14-hour composite was collected overnight at a sampling frequency of 15 min. In two prisons (PH and PDH), an intensified sampling setting (every 5 min) was tested between 07:00 and 09:00 to evaluate different strategies, with analysis indicating comparable SARS-CoV-2 data for both gene targets47. When composite sampling was not possible, grab samples were utilised, collecting one or two 1-litre samples based on water flow. Collected samples were transported on ice before storage at 4 °C until analysis, which occurred within 24 h of collection.

Wastewater parameters, including pH and electrical conductivity (EC), were measured using a multiparameter probe (Hach, USA) and a spectrophotometer (DR3900, Hach, USA). Total suspended solids (TSS) were determined according to Standard method 2540D (Rice et al., 2012) by filtering a defined water volume through a membrane, Whatman GF/F glass-fibre filter. Ammonia concentrations were measured using a kit (LCK 303– Hach, USA) following the manufacturer’s instructions.

### Additional data

Community wastewater data were obtained from the Environmental Monitoring for Health Protection (EMHP) programme led by the Joint Biosecurity Centre (part of NHS Test and Trace). Daily numbers of new COVID-19 cases among prisoners and staff were obtained from HMPPS. Each site reported anonymised symptomatic and asymptomatic cases to the project team. Twice-weekly testing of staff was introduced in November 2020. Uptake varied substantially between sites^9^, and prisoner testing was symptom-led unless public health professionals identified an outbreak (of linked cases). Wastewater data was compared with case data to enable weekly reporting of SARS-CoV-2 presence and trends with local and national data to inform local decision-making.

### Sample concentration and RNA extraction

Wastewater samples (200 mL) were processed as previously described^46^. Briefly, samples were centrifuged to remove large particles, and virus particles were concentrated with 50 mL of polyethene glycol (PEG) solution (40% PEG-800, 8% NaCl), shaken at 200 rpm for 15 min. Concentrates were acquired via successive rounds of centrifugation until residual PEG was removed. The resulting pellet was resuspended in an 8:1 mixture of TRIzol Reagent (Invitrogen, USA): chloroform, mixed thoroughly, and centrifuged at 12,000 x g for 15 min at 4 °C to separate RNA from other layers. RNA was further purified using the Nucleospin RNA kit (Macherey-Nagel, Germany), with modifications to exclude bead beating and lysis steps.

### Detection of SARS-CoV-2 by RT-qPCR

SARS-CoV-2 nucleocapsid (N), N1 and envelope (E) genes, along with Φ6, were quantified using reverse transcriptase-polymerase chain reaction (RT-qPCR) with the RNA UltraSense™ One-Step Quantitative RT-PCR System (ThermoFisher, UK) or the SsoAdvanced™ Universal Probes Supermix (Bio-Rad, USA). Analysis was conducted with QuantStudio™ 7 Pro or CFX C1000 System (Bio-Rad, USA) instruments. All samples and negative controls were run in duplicate. Primer/probe sequences were as follows: for N1^59^, forward 5′-GACCCCAAAATCAGCGAAAT-3′, reverse 5′-TCTGGTTACTGCCAGTTGAATCTG-3′ and probe 5′-FAM-ACCCCGCATTACGTTTGGTGGACC-BHQ1-3′; for E^60^, forward 5′-ACAGGTACGTTAATAGTTAATAGCGT-3′, reverse 5′-ATATTGCAGCAGTACGCACACA-3′ and probe 5′-HEX-ACACTAGCCATCCTTACTGCGCTTCG-BHQ1-3′, analysing duplicate RNA samples against negative controls. Quantification was based on cycle threshold (Ct) values against a standard curve generated from synthetic plasmids (Integrated DNA Technologies, USA). Synthetic RNA was spiked into each reaction to monitor for inhibition. Limits of detection (LOD) were 956 GC/L (N1) and 2401 GC/L (E); limits of quantification (LOQ) were 7859 GC/L (N1) and 18,138 GC/L (E), as determined by serial-dilution spike-in experiments. Positive detection required Ct values below the LOD threshold and no amplification in no-template controls. Average coefficients of variation were 23.48% (N1) and 25.38% (E), with an average Φ6 recovery of 8.49%.

The LOD refers to the lowest concentration of a target analyte (e.g., SARS-CoV-2 RNA fragments, drugs, or biomarkers) that can be reliably distinguished from background noise in the wastewater sample. In practical terms, this means the smallest signal that indicates the likely presence of the pathogen or compound in the prison population, though not necessarily at a level where precise quantification is possible.

The LOQ is the lowest concentration of the analyte that can be measured with acceptable accuracy and precision. In wastewater settings, this represents the point at which variation in viral RNA recovery, inhibitors in the wastewater matrix, and assay performance allow for meaningful estimation of concentration levels rather than simply presence/absence.

Two RT-qPCR platforms were used interchangeably; both quantified N1 and E with identical primer–probe assays, standards, and controls. LOD/LOQ were defined per assay and applied across instruments. Inter-instrument equivalence was evaluated by analysing replicate extracts on both platforms and observing concordant standard-curve parameters (slope, intercept, efficiency); no platform-specific correction was required.

### SARS-CoV-2 variant identification from prison wastewater

Pooled weekly samples were subjected to the detection of SARS-CoV-2 Variants of Concern or Variants under investigation (VOC/VUI). Samples underwent COVID-19 ARTIC v3 Illumina library construction and sequencing16 (Farr et al. 2020). This protocol involves generating cDNA from SARS-CoV-2 viral nucleic acid extracts and generating 400-nucleotide amplicons tiling the viral genome. The following VOC/VUI were screened: B.1.617.1 (Delta), B.1.617.2 (Delta), B.1.617.2_K417N (Delta), B.1.617.3 (Delta), B.1.1.7 (Alpha), B.1.1.7 + E484K (Alpha), B.1.351 (Beta), B.1.1.28.P1 (Gamma), AV.1, C.35.3, A.23.1, B.1.1.318, B.1.324.1, B.1.1.28.P3 (Zeta), B.1.1.28.P3 (Theta).

### Data and statistical analysis

All statistical analysis and data manipulation were performed in R 4.3.0.

#### Analysis of wastewater concentration levels and clinical testing

LOESS (Locally Estimated Scatterplot Smoothing) regression lines were used to depict trends in wastewater concentration levels. Correlations between daily/weekly clinical case counts and daily/weekly average SARS-CoV-2 concentrations in wastewater were quantified using Spearman’s rank correlation (ρ), appropriate for non-normal, potentially non-linear relationships. Because during a period of whole-population (mass) testing at one study prison, the number of PCR-positive individuals detected was approximately five times the routine case reports over the same interval, indicating substantial under-ascertainment, we applied a 5× correction factor to routine case counts only for sites/periods without mass testing to test for association.

#### Analysis of prison wastewater concentration and community wastewater concentration levels

For both prison and community wastewater data, a 7-day rolling average was calculated to adjust for non-collection days. For each prison, SARS-CoV-2 RNA concentrations recorded as zero were not excluded to ensure data were not skewed towards a higher mean. Non-detects were retained and treated as 0.5× LOD/LOQ prior to weekly aggregation. For each prison, we computed Spearman’s ρ between the prison series and the community series shifted from − 14 to + 14 days (1-day steps), and identified the lag with maximal ρ. For each lead/lag, Spearman’s correlation coefficient (ρ) was compared to the starting correlation coefficient on day zero to determine whether the strength of correlation improved. We summarised mean ρ and SD across prisons at each lag.

## Supplementary Information

Below is the link to the electronic supplementary material.

Supplementary Material 1

## Data Availability

The general datasets analysed during this study are available in the Newcastle University repository, via 10.25405/data.ncl.29627600. The sequencing datasets are available in the European Nucleotide Archive repository under study accession PRJEB95029.
