# Supplementary material for: Near-source wastewater surveillance as a non-invasive tool for disease detection in prisons
Source: Sci Rep. 2026 Jan 31;16:6815. doi: 10.1038/s41598-026-35801-1 (PMC12917126; doi:10.1038/s41598-026-35801-1)

**Supplementary information**

**Appendix Table 1: Average weekly clinical case and SARS-CoV-2 wastewater concentrations (GC/L) by prison.**

|  | **Week** | | | | | | | | | | | | | **P-value** | **rho** |
| --- | --- | --- | --- | --- | --- | --- | --- | --- | --- | --- | --- | --- | --- | --- | --- |
|  |  | **1** | **2** | **3** | **4** | **5** | **6** | **7** | **8** | **9** | **10** | **11** | **12** |  |  |
| **PNT** | **Cases** | 15 | 17 | 21 | 47 | 39 | 8 | 23 | 5 | 6 | 1 | 1 | 0 |  |  |
|  | **N1** | 1.97x10^5^ | 6.40x10^4^ | 4.31x10^4^ | 2.15x10^5^ | 6.14x10^5^ | 9.39x10^4^ | 9.68x10^3^ | 2.66x10^3^ | 0 | 0 | 0 | 0 | 0.001 | 0.824 |
|  | **E** | 1.16x10^6^ | 6.23x10^5^ | 2.13x10^5^ | 1.67x10^6^ | 1.53x10^6^ | 2.44x10^5^ | 7.37x10^4^ | 4.32x10^4^ | 0 | 6.64x10^3^ | 5.89x10^2^ | 3.24x10^3^ | 0.002 | 0.7986 |
| **PLN** | **Cases** | 0 | 7 | 3 | 2 | 2 | 0 | 0 | 0 | 0 | 0 | 1 | 0 |  |  |
|  | **N1** | 0 | 3.50x10^3^ | 8.21x10^2^ | 1.24x10^4^ | 8.89x10^2^ | 0 | 0 | 0 | 0 | 0 | 0 | 0 | 0.0001 | 0.8834 |
|  | **E** | 0 | 4.36x10^4^ | 0 | 1.53x10^5^ | 2.54x10^3^ | 0 | 0 | 0 | 7.53x10^1^ | 0 | 0 | 1.16x10^2^ | 0.094 | 0.505 |
| **PHH** | **Cases** | 21 | 21 | 15 | 12 | 13 | 0 | 1 | 1 | 0 | 0 | 0 | 0 |  |  |
|  | **N1** | 1.39x10^5^ | 2.52x10^5^ | 2.30x10^5^ | 3.71x10^5^ | 2.96x10^4^ | 6.67x10^3^ | 2.52x10^3^ | 6.97x10^1^ | 0 | 0 | 0 | 0 | 0.0006 | 0.841 |
|  | **E** | 1.06x10^6^ | 2.84x10^6^ | 2.20x10^6^ | 2.81x10^6^ | 6.02x10^5^ | 3.22x10^4^ | 3.18x10^4^ | 1.82x10^4^ | 3.60x10^3^ | 1.67x10^3^ | 8.87x10^2^ | 0 | 0.0004 | 0.8515 |
| **PFK** | **Cases** | 1 | 0 | 0 | 0 | 1 | 2 | 3 | 0 | 1 | 0 | 1 | 0 |  |  |
|  | **N1** | 0 | 0 | 0 | 0 | 3.11x10^1^ | 0 | 0 | 0 | 0 | 0 | 0 | 0 | 0.5536 | 0.190 |
|  | **E** | 0 | 0 | 0 | 0 | 0 | 0 | 0 | 0 | 0 | 0 | 2.72x10^3^ | 0 | 0.5536 | 0.1903 |
| **PDHA&B** | **Cases** | 55 | 126 | 108 | 13 | 9 | 7 | 5 | 0 | 2 | 0 | 1 | 0 |  |  |
|  | **N1** | 5.31x10^5^ | 3.31x10^6^ | 1.61x10^6^ | 1.31x10^6^ | 1.43x10^5^ | 2.05x10^4^ | 6.91x10^3^ | 0 | 0 | 0 | 0 | 0 | 4.535E-07 | 0.964 |
|  | **E** | 5.61x10^6^ | 3.94x10^7^ | 1.67x10^7^ | 1.04x10^7^ | 1.77x10^6^ | 1.08x10^5^ | 5.56x10^4^ | 0 | 0 | 0 | 0 | 0 | 4.535E-07 | 0.964 |
| **PDB** | **Cases** | 2 | 1 | 0 | 0 | 2 | 2 | 0 | 0 | 2 | 1 | 0 | 0 |  |  |
|  | **N1** | 0 | 0 | 0 | 0 | 0 | 5.76x10^2^ | 0 | 0 | 0 | 0 | 0 | 0 | 0.2212 | 0.381 |
|  | **E** | 0 | 0 | 0 | 0 | 0 | 1.25x10^3^ | 0 | 0 | 0 | 0 | 4.91x10^1^ | 0 | 0.7301 | 0.111 |
| **PWF** | **Cases** | 7 | 7 | 5 | 3 | 6 | 5 | 1 | 5 | 0 | 0 | 1 | 0 |  |  |
|  | **N1** | 1.39x10^4^ | 1.68x10^4^ | 3.20x10^3^ | 1.57x10^3^ | 5.71x10^3^ | 0 | 3.12x10^3^ | 7.69x10^2^ | 5.73x10^3^ | 1.74x10^3^ | 2.01x10^3^ | 0 | 0.1429 | 0.449 |
|  | **E** | 6.26x10^4^ | 1.98x10^5^ | 3.09x10^4^ | 1.11x10^4^ | 6.57x10^4^ | 8.61x10^3^ | 2.36x10^3^ | 1.21x10^3^ | 4.10x10^3^ | 0 | 2.67x10^3^ | 0 | 0.001 | 0.8165 |
| **PNH** | **Cases** | 1 | 0 | 2 | 1 | 0 | 1 | 0 | 0 | 0 | 0 | 0 | 0 |  |  |
|  | **N1** | 3.05x10^4^ | 4.24x10^3^ | 4.70x10^3^ | 1.85x10^3^ | 0 | 1.49x10^3^ | 1.72x10^3^ | 1.85x10^3^ | 0 | 1.11x10^3^ | 0 | 3.00x10^3^ | 0.0836 | 0.5193 |
|  | **E** | 2.82x10^5^ | 7.07x10^4^ | 1.31x10^3^ | 0 | 0 | 0 | 0 | 0 | 4.20x10^2^ | 1.03x10^3^ | 0 | 0 | 0.393 | 0.2728 |
| **PML** | **Cases** | 65 | 26 | 1 | 0 | 3 | 1 | 1 | 2 | 1 | 1 | 0 | 1 |  |  |
|  | **N1** | 1.29x10^6^ | 2.57x10^5^ | 1.22x10^5^ | 3.94x10^3^ | 1.05x10^4^ | 0 | 3.84x10^3^ | 0 | 6.31x10^3^ | 0 | 0 | 4.29x10^2^ | 0.06611 | 0.5463 |
|  | **E** | 8.69x10^5^ | 7.68x10^5^ | 1.59x10^5^ | 9.97x10^3^ | 1.81x10^4^ | 0 | 0 | 0 | 2.36x10^4^ | 5.60x10^3^ | 0 | 1.57x10^3^ | 0.888 | 0.512 |
| **PLH1&2** | **Cases** | 0 | 0 | 1 | 1 | 0 | 0 | 0 | 0 | 0 | 0 |  |  |  |  |
|  | **N1** | 1.53x10^3^ | 0 | 0 | 0 | 2.30x10^3^ | 1.25x10^3^ | 0 | 2.93x10^3^ | 5.71x10^3^ | 7.93x10^2^ |  |  | 0.108 | -0.5388 |
|  | **E** | 0 | 0 | 0 | 0 | 0 | 0 | 0 | 0 | 0 | 0 |  |  | N/A | N/A |
| **PHB** | **Cases** | 78 | 72 | 21 | 16 | 4 | 6 | 1 | 2 | 3 | 1 | 1 | 0 |  |  |
|  | **N1** | 1.06x10^4^ | 3.73x10^3^ | 2.59x10^3^ | 0 | 0 | 3.94x10^4^ | 0 | 2.12x10^2^ | 0 | 3.35x10^2^ | 0 | 2.46x10^2^ | 0.1126 | 0.482 |
|  | **E** | 1.87x10^4^ | 3.16x10^3^ | 0 | 0 | 0 | 3.61x10^4^ | 0 | 1.08x10^3^ | 0 | 3.05x10^3^ | 0 | 4.38x10^2^ | 0.368 | 0.286 |
| **PHO&N** | **Cases** | 20 | 56 | 24 | 2 | 0 | 1 | 0 | 0 | 0 | 0 | 0 | 0 |  |  |
|  | **N1** | 2.28x10^5^ | 4.99x10^5^ | 4.11x10^6^ | 5.95x10^7^ | 4.73x10^6^ | 1.36x10^6^ | 4.29x10^5^ | 1.16x10^5^ | 2.17x10^4^ | 5.64x10^3^ | 3.64x10^3^ | 1.33x10^3^ | 0.0618 | 0.554 |
|  | **E** | 8.08x10^5^ | 1.70x10^6^ | 8.55x10^6^ | 3.10x10^7^ | 4.20x10^6^ | 1.65x10^6^ | 3.75x10^5^ | 1.43x10^5^ | 2.98x10^4^ | 1.08x10^4^ | 2.44x10^3^ | 2.50x10^3^ | 0.0137 | 0.6862 |
| **PFS** | **Cases** | 1 | 13 | 0 | 0 | 0 | 0 | 0 | 0 | 1 | 0 | 1 | 0 |  |  |
|  | **N1** | 1.96x10^3^ | 1.32x10^3^ | 2.20x10^3^ | 0 | 1.17x10^3^ | 0 | 1.10x10^3^ | 0 | 0 | 2.59x10^3^ | 0 | 0 | 0.8899 | 0.0449 |
|  | **E** | 0 | 0 | 0 | 0.0 0 | 0 | 0 | 0 | 0 | 0 | 0 | 0 | 3.79x10^3^ | 0.5125 | -0.21 |

**Appendix Table 2: Wastewater characteristics by prison.**

|  | **Conductivity (µs)** | | | | **pH** | | | | **TSS (mg/L)** | | | | **NH4-N (mg/L)** | | | |
| --- | --- | --- | --- | --- | --- | --- | --- | --- | --- | --- | --- | --- | --- | --- | --- | --- |
| **Prison** | **Min** | **Max** | **Mean** | **SD** | **Min** | **Max** | **Mean** | **SD** | **Min** | **Max** | **Mean** | **SD** | **Min** | **Max** | **Mean** | **SD** |
| **PNT** | 595 | 1070 | 897.4 | 102.5 | 7.20 | 8.15 | 7.66 | 0.23 | 27 | 705 | 178.2 | 125 | 19.7 | 61 | 42.5 | 10.7 |
| **PLN** | 490 | 1888 | 1115 | 328.4 | 6.73 | 8.38 | 7.70 | 0.40 | 15 | 10338 | 1116 | 1880 | 11.4 | 78.4 | 41.9 | 18.95 |
| **PHH** | 545 | 998 | 772.7 | 78.5 | 6.00 | 8.51 | 7.00 | 0.57 | 31 | 3860 | 698.8 | 814.6 | 21.4 | 60.8 | 45.6 | 8.8 |
| **PFK** | 398 | 1298 | 867.2 | 165.6 | 6.25 | 8.60 | 7.52 | 0.42 | 16 | 442 | 156.5 | 95.54 | 14.7 | 60.9 | 44.29 | 11.9 |
| **PDHA&B** | 471 | 1584 | 859.6 | 228.9 | 6.74 | 8.88 | 8.38 | 0.50 | 24 | 3900 | 478.4 | 918.5 | 20.8 | 118.2 | 83.59 | 23.61 |
| **PDB** | 427 | 1347 | 793.4 | 242.6 | 6.78 | 10.25 | 7.99 | 0.53 | 8 | 2610 | 153.1 | 384.6 | 11.2 | 80.2 | 31.3 | 14 |
| **PWF** | 350 | 1139 | 651.3 | 148.7 | 6.70 | 8.50 | 7.70 | 0.49 | NA | NA | NA | NA | NA | NA | NA | NA |
| **PNH** | 390 | 4000 | 782.9 | 549.7 | 6.20 | 9.30 | 8.05 | 0.83 | NA | NA | NA | NA | NA | NA | NA | NA |
| **PML** | 1676 | 2686 | 1943 | 181.8 | 6.87 | 8.48 | 7.64 | 0.34 | 16 | 392 | 113.7 | 107.9 | 16.3 | 53.3 | 31.2 | 8.87 |
| **PLH1&2** | 690 | 2186 | 1138 | 285.4 | 6.00 | 9.40 | 7.66 | 0.83 | NA | NA | NA | NA | NA | NA | NA | NA |
| **PHB** | 8.07 | 5367 | 2384 | 874.8 | 5.45 | 8.36 | 7.19 | 0.75 | 32 | 12504 | 1436 | 3355 | 8.8 | 39.1 | 17.86 | 6.79 |
| **PHO&N** | 1180 | 8795 | 2783 | 923.1 | 5.44 | 8.93 | 7.45 | 0.67 | 60 | 14180 | 1246 | 1999 | 14 | 105.2 | 48.24 | 17.45 |
| **PFS** | 670 | 4233 | 2271 | 1355 | 7.10 | 8.40 | 7.77 | 0.36 | NA | NA | NA | NA | NA | NA | NA | NA |

**Appendix Figure 1: *Summary of change in SARS-CoV-2 RNA concentration over time*, using N1 and E gene targets for the study.** The darker the shading the higher the viral presence.
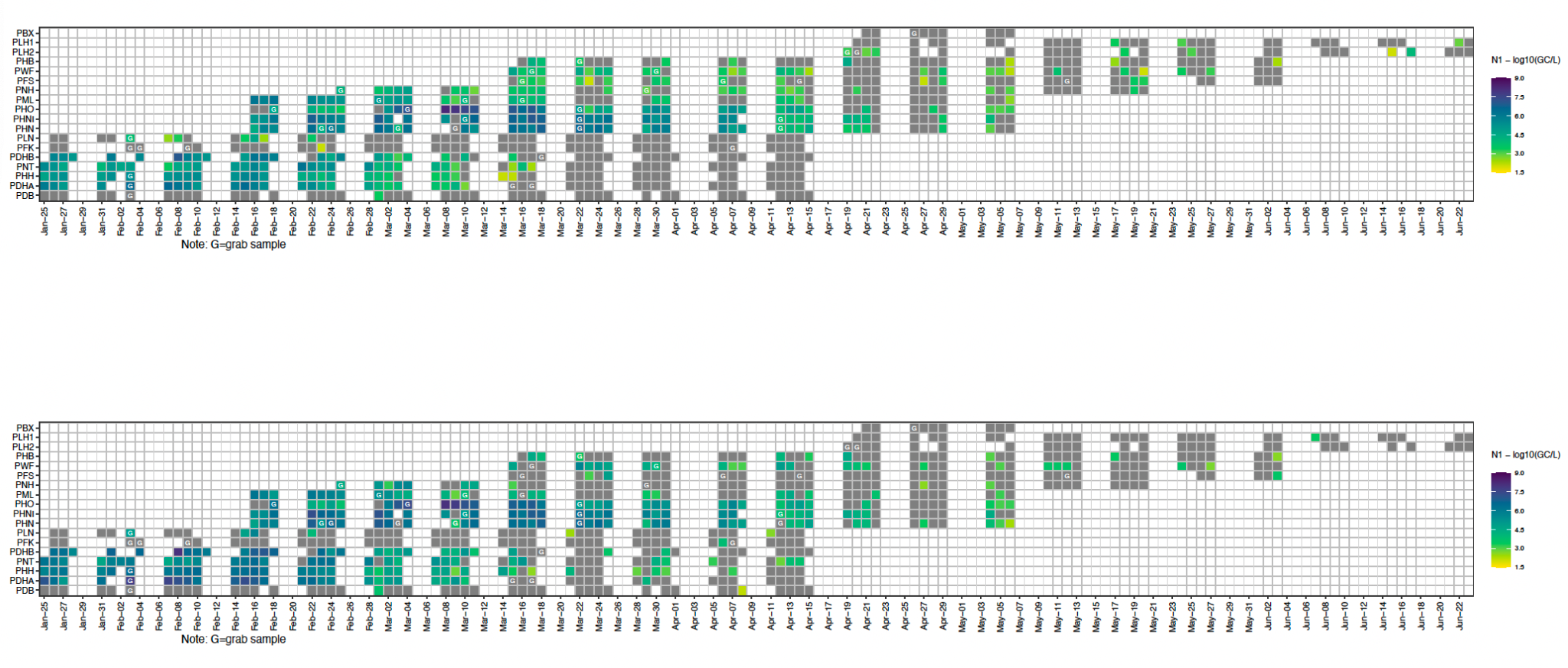


**Appendix Figure 2: Lead/lag analysis of SARS-CoV-2 concentration levels in prison and community wastewater.** The grey shadow represents the standard deviation. The dashed red line indicates the time when the correlation coefficient is highest.


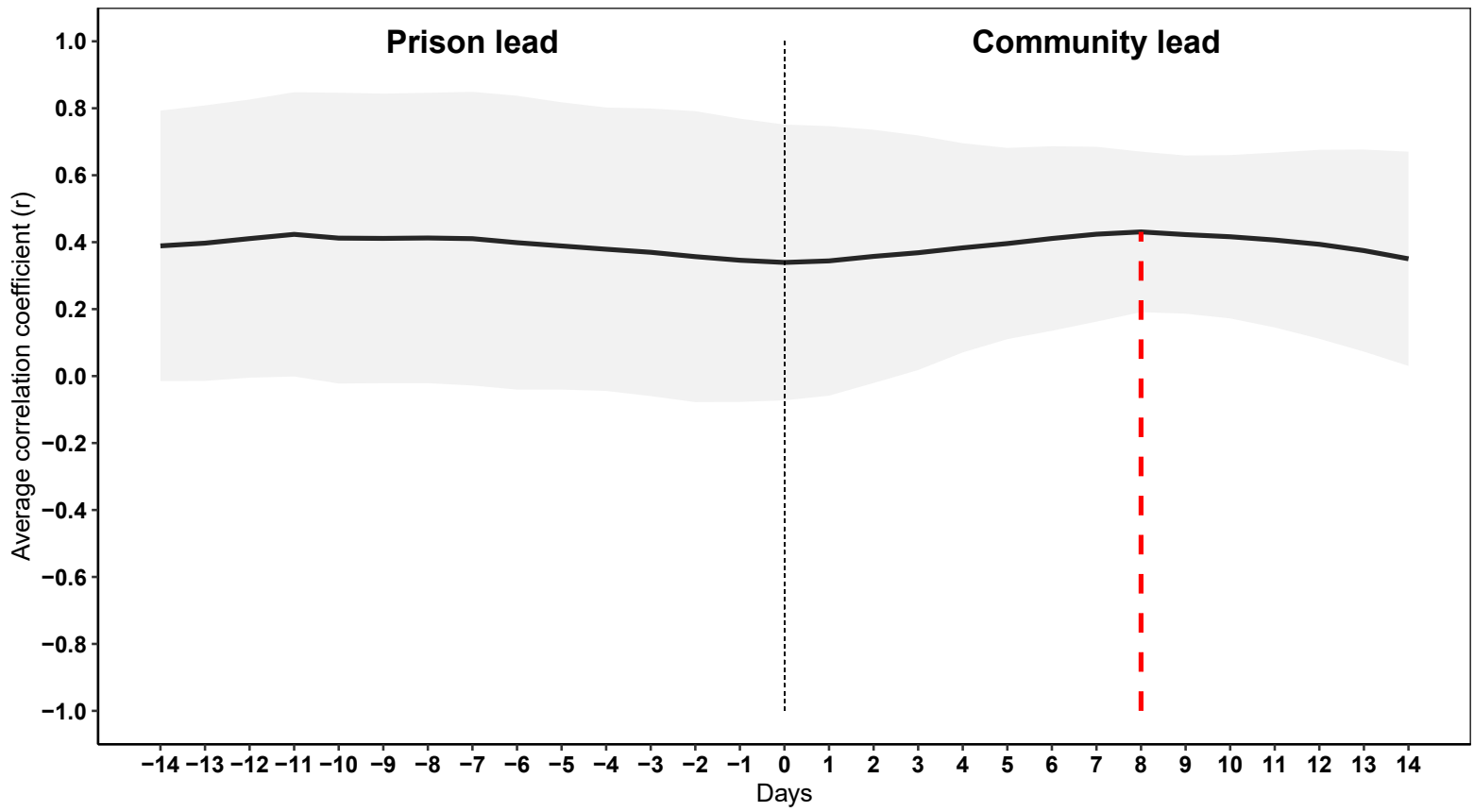


**Appendix Figure 3: Lead/lag analysis of SARS-CoV-2 levels comparing prison and community wastewater data.** Of the 13 sites (11 prisons), SARS-CoV-2 was identified and elevated in seven prisons, including two women-only sites, two adult male category C, an adult male category A, and two local prisons category B before detection in community wastewater. The findings highlight the interaction between prisons and their local community through staff, visitors, and prisoners mixing directly and indirectly. The grey shadow represents the standard deviation. The dashed red line indicates the time when the correlation coefficient is highest.


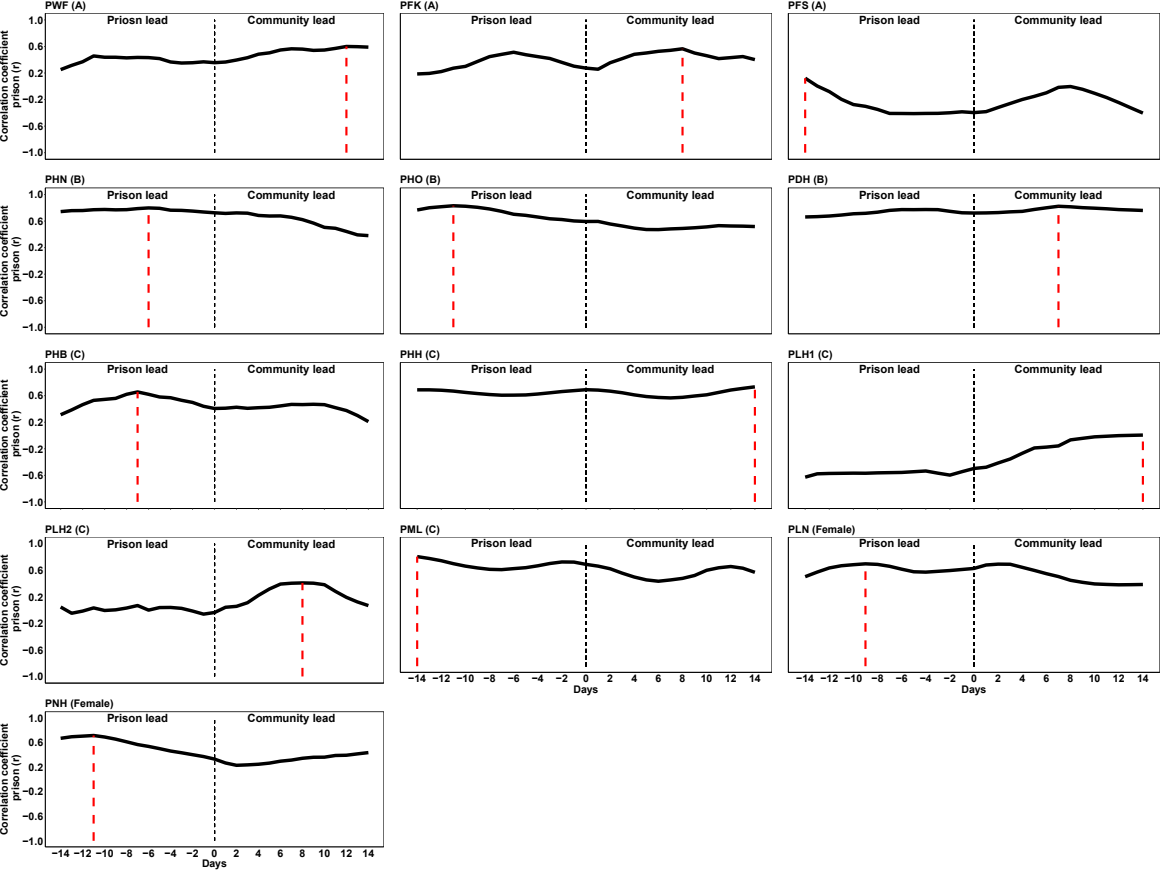

Supplement: Supplementary file 1 — Supplementary Material 1 [file 41598_2026_35801_MOESM1_ESM.docx]
